# Glyceollin I Reverses Epithelial to Mesenchymal Transition in Letrozole Resistant Breast Cancer through ZEB1

**DOI:** 10.3390/ijerph13010010

**Published:** 2015-12-22

**Authors:** Patrick P. Carriere, Shawn D. Llopis, Anna C. Naiki, Gina Nguyen, Tina Phan, Mary M. Nguyen, Lynez C. Preyan, Letitia Yearby, Jamal Pratt, Hope Burks, Ian R. Davenport, Thu A. Nguyen, KiTani Parker-Lemieux, Florastina Payton-Stewart, Christopher C. Williams, Stephen M. Boué, Matthew E. Burow, Bridgette Collins-Burow, Aaron Hilliard, A. Michael Davidson, Syreeta L. Tilghman

**Affiliations:** 1 College of Pharmacy, Xavier University of Louisiana, 1 Drexel Drive, New Orleans, LA 70125, USA; pcarriere@msm.edu (P.P.C.); sllopis@xula.edu (S.D.L.); acnaiki@gmail.com (A.C.N.); gi.nguye@gmail.com (G.N.); tphan0917@gmail.com (T.P.); marym.nguy@gmail.com (M.M.N.); lynezpreyan@gmail.com (L.C.P.); lyearby@xula.edu (L.Y.); jpratt1@xula.edu (J.P.); tnguye71@xula.edu (T.A.N.); kparker1@xula.edu (K.P.-L.); cwillia35@xula.edu (C.C.W.); 2 Tulane University Health Sciences Center, 1430 Tulane Ave, New Orleans, LA 70112, USA; hburks@tulane.edu (H.B.); mburow@tulane.edu (M.E.B.); bcollins1@tulane.edu (B.C.-B.); 3 Division of Biological and Public Health Sciences, College of Arts and Sciences, Xavier University of Louisiana, 1 Drexel Drive, New Orleans, LA 70125, USA; idavenpo@xula.edu; 4 Division of Mathematical and Physical Sciences, College of Arts and Sciences, Xavier University of Louisiana, 1 Drexel Drive, New Orleans, LA 70125, USA; flpayton@xula.edu; 5 Southern Regional Research Center, United States Department of Agriculture, New Orleans, LA 70124, USA; steve.boue@ars.usda.gov; 6 Division of Basic Sciences, College of Pharmacy and Pharmaceutical Sciences, Florida A&M University, 1415 S. Martin L. King Jr. Blvd., Tallahassee, FL 32307, USA; aaron.hilliard@famu.edu (A.H.); michael.davidson@famu.edu (A.M.D.)

**Keywords:** letrozole resistance, epithelial mesenchymal transition, breast cancer, phytochemicals, aromatase inhibitors, metastasis

## Abstract

Although aromatase inhibitors are standard endocrine therapy for postmenopausal women with early-stage metastatic estrogen-dependent breast cancer, they are limited by the development of drug resistance. A better understanding of this process is critical towards designing novel strategies for disease management. Previously, we demonstrated a global proteomic signature of letrozole-resistance associated with hormone-independence, enhanced cell motility and implications of epithelial mesenchymal transition (EMT). Letrozole-resistant breast cancer cells (LTLT-Ca) were treated with a novel phytoalexin, glyceollin I, and exhibited morphological characteristics synonymous with an epithelial phenotype and decreased proliferation. Letrozole-resistance increased Zinc Finger E-Box Binding Homeobox 1 (ZEB1) expression (4.51-fold), while glyceollin I treatment caused a −3.39-fold reduction. Immunofluorescence analyses resulted of glyceollin I-induced increase and decrease in E-cadherin and ZEB1, respectively. *In vivo* studies performed in ovariectomized, female nude mice indicated that glyceollin treated tumors stained weakly for ZEB1 and N-cadherin and strongly for E-cadherin. Compared to letrozole-sensitive cells, LTLT-Ca cells displayed enhanced motility, however in the presence of glyceollin I, exhibited a 68% and 83% decrease in invasion and migration, respectively. These effects of glyceollin I were mediated in part by inhibition of ZEB1, thus indicating therapeutic potential of glyceollin I in targeting EMT in letrozole resistant breast cancer.

## 1. Introduction

Less than a decade ago the aromatase inhibitor, letrozole, was approved for adjuvant treatment of postmenopausal women with estrogen receptor (ER) positive, early stage breast cancer. Despite the significant improvements in the outcome of breast cancer that is ER-positive following letrozole treatment, a significant percentage (ranging from 30% to 65%) of patients either do not respond to aromatase inhibitors [1] or develop resistance and relapse after continued use [2,3,4]. To date, few studies have documented potential therapeutic strategies for letrozole refractory tumors beyond cytotoxic chemotherapy, and those include either trastuzumab, intermittent letrozole treatment [5,6,7] and histone deacetylase (HDAC) inhibitors [3,8]. However, one of the major obstacles associated with these strategies is that eventually the tumors continue to proliferate, thereby creating a critical need to identify mechanism(s) of resistance and ultimately develop potential therapeutic options. While current therapeutic strategies aimed at treating letrozole refractory tumors are targeted towards inhibition of growth factor receptor signaling pathways, our group recently demonstrated that as cells transition to letrozole resistance, cellular motility is enhanced [9]. These findings may represent an opportunity for alternative approaches to treat letrozole resistant breast cancer.

Breast cancer metastasis accounts for the majority of deaths from breast cancer. While metastatic breast cancer (MBC) is currently incurable in most patients, those with hormone receptor positive disease do benefit from endocrine therapy. However, despite the availability of endocrine agents such as aromatase inhibitors (*i.e*., letrozole, anastrozole and exemestane) and antiestrogens (*i.e*., tamoxifen), some tumors may exhibit *de novo* resistance while others will eventually become resistant to endocrine therapy, resulting in disease progression. One potential mechanism for metastatic spread is the epithelial to mesenchymal transition (EMT) [10]. Having recently demonstrated a potential role for EMT in letrozole resistance we were interested in defining key factors involved in this process. It has been shown that the zinc finger E-box binding homeobox 1 (ZEB1) transcription factor plays a critical role in EMT in breast cancer [11,12,13,14]. As it is becoming increasingly more critical to better understand the molecular pathways contributing to metastasis and endocrine resistance we chose to explore the role of various canonical EMT markers including ZEB1 and the loss of E-cadherin in letrozole resistance.

Many naturally occurring agents, particularly bioactive compounds present in plants, have recently gained interest as potential therapeutics for breast cancer. Increasing epidemiological studies regarding consumption of dietary soy provides a rationale for various nutritional strategies designed to contribute to breast cancer prevention [15,16] and the flavonoid family of soy-derived phytochemicals, particularly glyceollins, has been implicated for the prevention and potential treatment of carcinogen-induced mammary tumorigenesis [17]. Additionally, glyceollins play key roles in inhibiting angiogenesis [18,19] and inflammation [20]. Glyceollins, a group of novel phytoalexins consisting of three isomers (I, II and III), were isolated from activated soy, and demonstrated to be novel antiestrogens that bind to the ER and inhibit estrogen-induced tumor progression [21]. Previously glyceollin I was identified as the most active component of the combined glyceollin mixture [22]. Glyceollin I exhibited potent antiestrogenic properties in estrogen-dependent cells by inhibiting ER-mediated gene expression, cell proliferation and survival. While it has been demonstrated that glyceollins are novel antiestrogens, an alternant mechanism has been suggested, whereby glyceollins target ER‑independent pathways regulating tumor cell proliferation and/or survival of triple negative breast cancer cells [23]. The biological activity of glyceollin I and its underlying mechanisms of action in regard to letrozole-resistant breast cancer *in vitro* and *in vivo* is largely unknown. Therefore, since letrozole-resistant tumors no longer require estrogen for growth we chose to investigate whether glyceollins could alter similar pathways involved in regulating tumorigenesis and metastasis.

## 2. Materials and Methods

### 2.1. Cell Culture

Human AC-1 breast cancer cells (MCF-7 cells stably transfected with the human aromatase gene) were kindly provided by Dr. Angela Brodie and were cultured in 75-cm^2^ flasks in DMEM (Invitrogen, Waltham, MA, USA) supplemented with 5% fetal bovine serum (FBS), penicillin-streptomycin, antimycotic-antibiotic (10,000 U/mL penicillin G sodium; 10,000 μg/mL streptomycin sulfate; and 25 μg/mL amphotericin B (Fungizone), and 7.5 μg/mL geneticin (Invitrogen). Human LTLT-Ca cells (long-term letrozole treated MCF-7 cells stably transfected with the human aromatase gene) were generously provided by Dr. Angela Brodie and were cultured in 75-cm^2^ flasks in phenol red-free IMEM (Invitrogen) supplemented with 10% charcoal-stripped fetal bovine serum (CS-FBS), penicillin-streptomycin, antimycotic-antibiotic (10,000 U/mL penicillin G sodium; 10,000 μg/mL streptomycin sulfate); and 25 μg/mL amphotericin B (Fungizone), 7.5 μg/mL geneticin (Invitrogen) and 1 μM letrozole (Sigma). The culture flasks were maintained in a tissue culture incubator in a humidified atmosphere of 5% CO_2_ and 95% air at 37 °C. The LTLT-Ca cells were isolated from tumors of aromatase transfected MCF-7 cells grown in ovariectomized nude mice following 56 weeks of treatment with letrozole. After long-term letrozole treatment, the tumors acquired the ability to proliferate in the presence of the drug. Tumors were then removed and grown in culture in the presence of letrozole [24]. Both AC-1 and LTLT-Ca cells are derivatives of the MCF-7 cell line and were authenticated by short tandem repeat profiling from ATCC and results verified both cell lines shared greater than 85% homology with the MCF-7 cell line. Cell lines with ≥80% match are considered to be related (*i.e*., derived from a common ancestry). In brief seventeen short tandem repeat (STR) loci plus the gender determining locus, Amelogenin, were amplified using the commercially available PowerPlex^®^ 18D Kit (Promega). The cell line samples were processed using the ABI Prism^®^ 3500xl Genetic Analyzer. Data were analyzed using GeneMapper^®^ ID-X v1.2 software (Applied Biosystems). Appropriate positive and negative controls were run and confirmed for each sample submitted. Cell lines were authenticated using Short Tandem Repeat (STR) analysis as described in 2012 in ANSI Standard (ASN-0002) by the ATCC Standards Development Organization (SDO) and in Capes-Davis *et al.* [25].

### 2.2. Proliferation Assays

Proliferation assays were performed as previously described [26]. Specifically, the AC-1 and LTLT-Ca cells were plated in 96-well plates at a density of 1 × 10^3^ cells per well for each cell line and allowed to recover for 24 h. Pharmacological treatments were added containing either the vehicle or 10 μM glyceollin I in four wells each. The alamarBlue dye (Life Technologies, Grand Island, NY, USA) was added to each well at 10% of the total volume and measured every week for three weeks. Biotek Synergy 4 Plate Reader was used to measure absorbance and background wavelengths at 550 nm and 630 nm to determine proliferation and calculated as a percent of the controls as follows:

Antiproliferative activity = [Absorbance of viable cells (control) − Absorbance of viable cells (treated)]/Absorbance of viable cells (control)

### 2.3. Colony Formation Assays

Cells were cultured in standard growth media and seeded at a density of 1000 cells/well in a 6 well plate. The cells were allowed to attach overnight and treated on the following day with DMSO vehicle, androstenedione, tamoxifen and glyceollin I. Media was replaced every 7 days and treated with appropriate drug for 3 weeks. After 3 weeks the media was removed and the cells were fixed with formaldehyde and dried overnight. The cells were then washed and stained with crystal violet and dried. Colonies were counted.

### 2.4. Cell Migration and Invasion Assays

Matrigel (BD Biosciences, San Jose, CA, USA) solution was prepared and diluted into serum-free media and used to coat the inner chamber of a 24-well ThinCert^TM^ cell culture inserts (for cell invasion assays only). The insert was incubated and washed with serum-free media. The cells were seeded into a 25 cm^2^ flask with appropriate media and treated with DMSO control or 10 μM glyceollin I for 24 h. On the following day, the cells were trypsinized, centrifuged and resuspended in serum-free media. Afterwards 250 μL of 2.5 × 10^4^ cells were added to the inner chamber of the insert and 500 μL of either serum-free or serum-containing media was added to the outer chamber. Cells were incubated for 24 h (migration) 48 h (invasion) at 37° with 5% CO_2_. The media was aspirated and the cells in the bottom of the well were fixed with 4% formaldehyde for 10 min at room temperature. The formaldehyde was aspirated and stained with crystal violet in 0.5% methanol. Media was aspirated and cells were re-suspended in serum-free media to same cell concentration. The upper membrane was scraped to remove nonmigratory cells. The membrane was fixed between glass microscope cover slip using ProLong Gold (Life Technologies, Grand Island, NY, USA). All experiments were performed with *n* ≥ 6 and a total of 3 biological replicates were performed. The images were captured by microscopy using Olympus BX41 at 10X magnification. Cells were counted using ImageJ software.

### 2.5. Gel Electrophoresis and Western Blot Analysis

Equal amounts of total proteins (50 μg) was subjected to sodium dodecyl sulfate-polyacrylamide gel electrophoresis (SDS-PAGE) using the Mini-PROTEAN Tetra Cell electrophoresis module assembly (Bio-Rad, Hercules, CA, USA) and transferred at 4 °C overnight to nitrocellulose membranes (Bio-Rad). Immunodetections were performed using anti-rabbit antibodies against human ERα (Santa Cruz Biotechnology, Santa Cruz, CA, USA), ZEB1 (Santa Cruz Biotechnology, Santa Cruz, CA, USA), and GADPH (Sigma-Aldrich, St. Louis, MO, USA). Immunoreactive bands were visualized using the enhanced chemiluminescence detection reagents according to the manufacturer’s instructions and quantitated by densitometry using ImageJ software.

### 2.6. Quantitative RT-PCR

LTLT-Ca cells were plated at approximately 80% confluency in 100 mm culture dishes in 10% CS-IMEM and allowed to adhere overnight in a 37 °C incubator. The following day cells were transfected with 5 μg of EX-eGFP vector or CMV-eGFP-ZEB1 (GeneCopoeia, Rockville, MD, USA) plasmids using Lipofectamine 2000 transfections reagent (Invitrogen) per manufacturer’s instructions. After 24 h, cells were treated with vehicle or 10 μM glyceollin I then harvested 48 h after transfection. LTLT-Ca-vector and LTLTCa-ZEB1 cells were harvested for total RNA extraction using the Quick-RNA Miniprep kit (Zymo Research, Irvine, CA, USA) per manufacturer’s protocol. Quantity and quality of the RNA was determined by absorbance at 260 and 280 nm using the NanoDrop ND-1000 spectrophotometer. 1 μg of total RNA was reverse-transcribed using the iScript kit (BioRad Laboratories, Hercules, CA, USA) and qPCR was performed using SYBR-green (Bio-Rad Laboratories, Hercules, CA, USA). β-Actin, E-cadherin, N-cadherin and ZEB1 genes were amplified *n* > 3. Data was analyzed by comparing relative target gene expression to β-actin. Relative gene expression was analyzed using 2^−ΔΔCt^ method.

### 2.7. Gene Expression Superarrays

AC-1 cells were seeded into 75-cm^2^ flasks in DMEM supplemented with 5% FBS and LTLT-Ca cells were seeded into 75-cm^2^ flasks in phenol red-free IMEM supplemented with 5% CS-FBS. Total RNA was extracted from untreated AC-1 and LTLT-Ca cells ± glyceollin I. Each array profiles the expression of a panel of 96 genes. For each array, 2 μg of RNA was reverse-transcribed into cDNA in the presence of gene-specific oligonucleotide primers as described in the manufacturer’s protocol. cDNA template was mixed with the appropriate ready-to-use PCR master mix. Equal volumes were measured (in aliquots) into each well of the same plate, and then the real-time PCR cycling program was run. Quantitative RT-PCR was performed using the manufacturer’s protocols for the Human Cell Motility and Human Epithelial to Mesenchymal Transition (EMT) RT^2^ Profiler PCR Array (Qiagen). Relative gene expressions were calculated by using the 2^−ΔΔCt^ method, in which Ct indicates the fractional cycle number where the fluorescent signal reaches detection threshold. The “delta-delta” method (described by Pfaffl, 2001) uses the normalized ΔCt value of each sample, calculated using a total of five endogenous control genes (18S rRNA, HPRT1, RPL13A, GAPDH, and ACTB) [27]. Fold change values are then presented as average fold change = 2^−(average ΔΔCt)^ for genes in treated relative to control samples. Clinical variables were characterized using descriptive statistics, and the statistical significance of differences in gene expression between groups was calculated using Student’s *t* test. Fold changes greater than 2 were considered significant. All experiments were performed with a minimum of 3 biological replicates.

### 2.8. Immunofluorescence

LTLT-Ca cells were seeded in 8-well chamber slides (Thermo Scientific) pre-coated with 2% gelatin and grown to 50% confluence. Cells were fixed with in 4% formaldehyde and rinsed with PBS. The cells were permeabilized with 0.5% NP-40/PBS for 15 min and rinsed 3 times with PBS. The samples were incubated with 10% goat serum (Invitrogen Life Technologies) in PBS for 1 h at room temperature. Primary antibody 1:200 rabbit anti-E-cadherin (Cell Signaling Technology, Danvers, MA, USA), 1:200 ZEB1 (Cell Signaling Technology, Danvers, MA, USA), 1:200 Ki67 (Santa Cruz Biotechnology) was added to 10% goat serum/0.5% NP-40/PBS and incubated at 4° overnight. This was followed by 3 × 5 min washes in 1% goat serum/PBS and then addition of secondary antibodies Alexa Fluor goat anti-rabbit-488 (Invitrogen Life Technologies, Carlsbad, CA, USA) at 1:200 in 10% goat serum for 2 h at room temperature away from light. The samples were washed with 1% goat serum/PBS and stained with 300 nM DAPI (Invitrogen, Life Technologies) for 10 min using dark incubation. The coverslips were mounted on slides using ProLong Gold Antifade (Invitrogen Life Technologies) and imaged using an Olympus BX41 microscope (Olympus, Center Valley, PA, USA). The images were captured with a DP72 CCD driven by DP2 software (Olympus) and the color images were combined in the ImageJ software. All images were captured using the same exposure time for each channel and the similar results were observed in single primary antibody stained cells. Cells that were not stained had no observable background fluorescence. All immunofluorescence experiments were performed with a minimum of tumors from 3 separate mice with a minimum of 3 technical repeats.

### 2.9. Animal Xenograft Studies

Nu/nu immune-compromised female ovariectomized mice (29–32 days old) were obtained from Charles River Laboratories (Wilmington, MA, USA). The animals were allowed a period of adaptation in a sterile and pathogen-free environment with phytoestrogen-free food and water *ad libitum*. LTLT-Ca cells in the exponential phase of growth were harvested using PBS/EDTA solution and washed. Viable cells (5 × 10^6^) in a 50 µL sterile PBS suspension were mixed with 100 µL Matrigel Reduced Factors (BD Biosciences, Bedford, MA, USA). LTLT-Ca cells were injected in the mammary fat pad. All the procedures in animals were carried out under anesthesia using a ketamine/xylazine mixture. Tumors were allowed to form over 11 days and mice were randomized to two treatment groups with 4 mice per group: control (Con) and glyceollin mixture. The glyceollin mixture was suspended in a solution of DMSO (one-third volume) and PBS (two-third volume) and was given i.p. at 20 mg/kg/mouse/d for 40 days starting after tumors were measureable. Control was injected with vehicle daily for 40 days. At necropsy, animals were euthanized by exposure to a CO_2_ chamber. Tumors, uteri, brain, livers, and lungs were removed and either frozen in liquid nitrogen or fixed in 10% formalin for further analysis. All procedures involving these animals were conducted in compliance with State and Federal laws, standards of the U.S. Department of Health and Human Services, and guidelines established by the Xavier University of Louisiana University Animal Care and Use Committee. The facilities and laboratory animal program of Xavier University of Louisiana is accredited by the Association for the Assessment and Accreditation of Laboratory Animal Care.

### 2.10. Immunohistochemistry

Tissues were fixed in 4% neutral buffered formalin and dehydrated via an ethanol series. They were embedded in paraffin and sectioned at 7 µm. Sections were deparaffinization using Histo-Clear and the samples rehydrated through an ethanol series. Tissues were examined from a minimum of 3 mice per treatment group for three different proteins: E cadherin, N-cadherin and ZEB1. Heat-induced antigen retrieval was conducted using Tris-EDTA buffer pH 9 with 0.05% Triton X 100 in a pressure cooker for 20 min. Antibody dilutions were as follows: Anti-E cadherin (mouse monoclonal) 5 µg/mL, anti-N-cadherin (rabbit polyclonal) 7.5 µg/mL and Anti-AREB6 (rabbit polyclonal) 1:250. Staining was carried out using EXPOSE mouse and rabbit specific HRP/DAB detection IHC kit (Abcam, Cambridge, MA, USA). Counter staining with hematoxylin for 3 min and blueing with 0.2% ammonia water for 1 min. Slides were dehydrated using graded ethanol, two washes with Histo-Clear and mounted with Permount. Immunohistochemistry staining data represented as a semiquantitative Histo-score where the fractions of negative (score 0), weakly positive (score 1), positive (score 2), strongly positive (score 3) and very strongly positive (score 4) [28,29]. All sections had a negative control slide (no primary antibody) of an adjacent section to preclude nonspecific staining. The slides were imaged using an Olympus BX41 microscope (Olympus, Center Valley, PA) and images were captured with a DP72 CCD driven by DP2 software (Olympus).

### 2.11. Statistical Analysis

Data were summarized as the mean ± standard error of the mean (SEM) using the Graph Pad Prism V.6 software program. Analysis of variance models were employed to compare relative cell proliferation between control *versus* glyceollin I. A Tukey’s multiple comparisons post-test was performed to compare differences between groups where a *p* value < 0.05 was considered significant. A student *t* test was employed to compare relative gene expression between AC-1 cells *versus* LTLT-Ca cells and LTLT-Ca control *versus* LTLT-Ca + glyceollin I as well as cell motility between LTLT-Ca control *versus* LTLT-Ca + glyceollin I. Results are expressed as the mean unit ± SEM (**** *p* < 0.0001, *** *p* < 0.001, ** *p* < 0.01, * *p* < 0.05).

## 3. Results

### 3.1. Glyceollin I Inhibits Proliferation and Viability of Letrozole-Resistant Breast Cancer Cells

**Figure 1:**
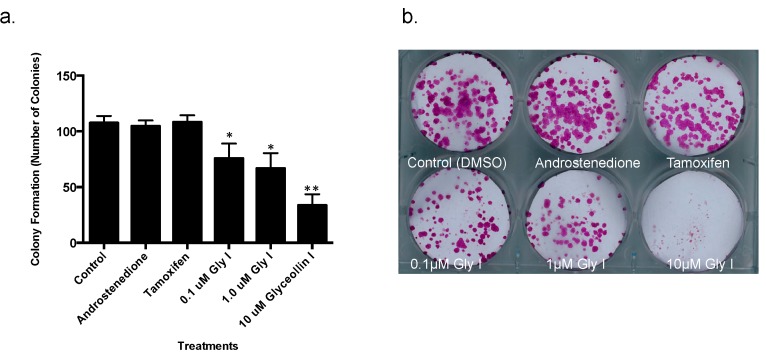

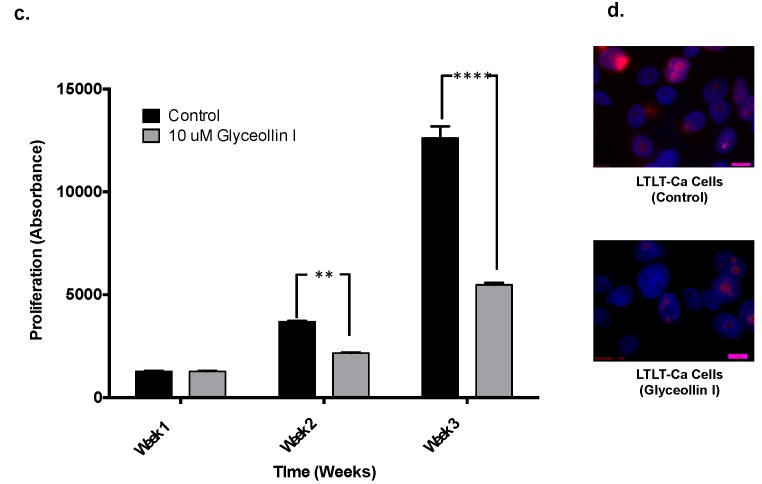
(**a**,**b**) Glyceollin I decreases the viability of letrozole resistant breast cancer cells. Letrozole resistant (LTLT-Ca) cells were treated with control (DMSO), androstenedione, tamoxifen and 0.1 μM, 1.0 μM and 10 μM glyceollin I and cell viability studies were performed. Graph depicts the percentage of cells that formed colonies after 3 weeks; (**c**) Glyceollin I decreases the proliferation of LTLT-Ca cells. In brief, cells were treated with control (DMSO) or 10 μM glyceollin I and long-term proliferation studies were conducted. The proliferation was measured using the alamarBlue assay and graph represents the proliferation (absorbance) of LTLT-Ca cells over the course of 3 weeks. Results are expressed as the mean unit ± SEM (**** *p* < 0.0001; *** *p* < 0.001; ** *p* < 0.01; * *p* < 0.05) of three independent experiments in triplicate; (**d**) Representative images of LTLT-Ca cell staining with anti-Ki67 (red staining) and DAPI (blue nuclear staining) in the presence and absence of 10 μM glyceollin I.

It has been reported that aromatase inhibitor resistant tumors are hormone refractory, estrogen-independent and become dependent on alternate signaling pathways for survival [9]. While the majority of our previous studies report the antiestrogenic properties of glyceollins, glyceollin can modestly inhibit tumor formation in estrogen-independent breast cancer cells [23]. Since glyceollin I is the most active component of the mixture [22], we chose to focus our efforts on its role in letrozole refractory breast cancer. Therefore, we tested the effect of glyceollin I on the proliferation and viability of LTLT-Ca cells using the colony formation assay, alamarBlue proliferation assay and Ki67 staining respectively. Glyceollin I treatment (10 μM) demonstrates a 68.74% ± 9.22% decrease in LTLT-Ca colony formation while the ER antagonist tamoxifen has no effect on cell viability (Figure 1a,b). Since the most effective dose was 10 μM we chose to continue our studies using this concentration. To rule out that this concentration was toxic, long-term proliferation studies were performed *in vitro*. Interestingly, glyceollin I treatment displayed antiproliferative effects and caused a time-dependent decrease in the growth of the letrozole resistant cells. As shown in Figure 1c, this effect persisted over the course of 3 weeks. Taken together, glyceollin I demonstrated both time dependent decreases in proliferation and a dose-dependent decrease in viablity. Consistent with the effect of glyceollin I on cell growth *in vitro*, proliferation (assessed by Ki67 staining) of tumor cells was significantly decreased (Figure 1d) validating that cells were still viable in the presence of 10 μM glyceollin I.

### 3.2. Glyceollin I Alters the Morphology of Letrozole-Resistant Breast Cancer Cells without Affecting ER Status

**Figure 2:**
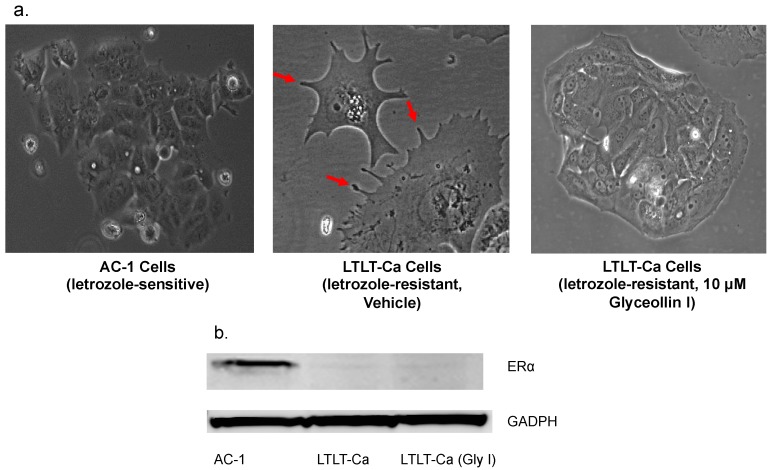
(**a**) Glyceollin I alters the cell morphology. The morphology of letrozole sensitive (AC-1) cells were compared to that of LTLT-Ca cells ± 10 μM glyceollin I. Cell images were captured by microscopy using Olympus BX41 at 10x magnification and red arrows indicate invadopodia; (**b**) Glyceollin I does not restore ER levels. AC-1, LTLT-Ca and LTLT-Ca cells treated with 10 μM glyceollin I were assayed by immunoblot to examine the expression of ERα and GAPDH (loading control). Representative immunoblot depicts that AC-1 cells are positive for ERα, while LTLT-Ca cells ± 10 μM glyceollin I express low ERα.

Recently, our group demonstrated that as breast cancer cells progress towards a letrozole resistant phenotype, they exhibit increased cell motility and invasiveness. As such, once cells become resistant to letrozole they transition from a round, uniform cell morphology to a less organized cell structure with protrusions of the plasma membrane [9]. To assess whether glyceollin I had the potential to convert the LTLT-Ca cells to a less invasive phenotype, LTLT-Ca cells were treated with glyceollin I and the morphology was compared to vehicle-treated LTLT-Ca cells and letrozole sensitive AC-1 cells. Glyceollin I enhanced the conversion of LTLT-Ca cells from a mesenchymal morphology (cell protrusions) to a more epithelial morphology (rounded) similar to that of the AC-1 cells (Figure 2a). Previously, we demonstrated that as cells become resistant to letrozole, there is a concomitant decrease in ERα and aromatase protein expression [9,30]. Since glyceollin I altered the morphology of the LTLT-Ca cells we were interested in determining whether ERα levels could be restored to those of the parental estrogen-dependent AC-1 cells. Western blot analyses were performed and results indicated that ERα expression remained low in the presence of glyceollin I, suggesting that cell remodeling may not be soley mediated through classical estrogen signaling pathways (Figure 2b).

### 3.3. Glyceollin I Alters EMT Marker Expression in LTLT-Ca Cells in Vitro

**Figure 3:**
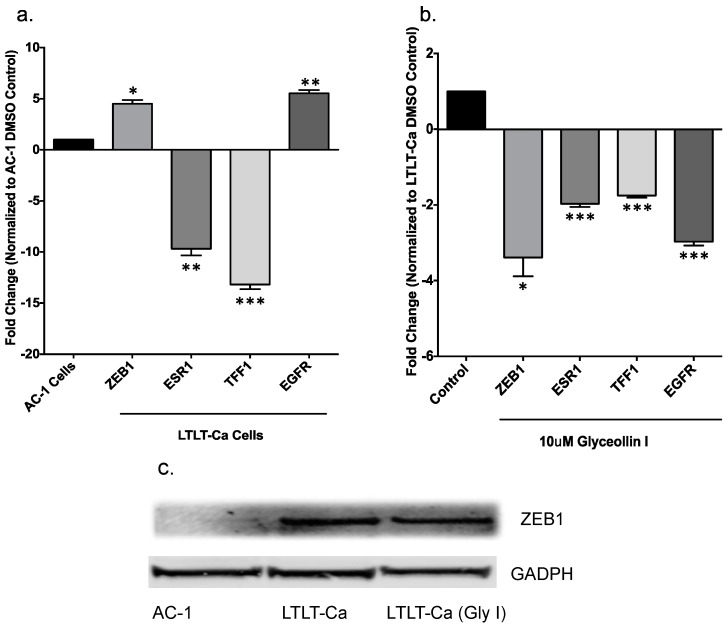
Glyceollin I alters EMT marker expression in LTLT-Ca cells. Total RNA was isolated from (**a**) AC-1 and LTLT-Ca cells or (**b**) LTLT-Ca cells treated with Glyceollin I (10 μM) and reverse transcribed into cDNA and subjected to real time RT-PCR analysis for quantitation of ESR1, ZEB1, TFF1 or EGFR. Results are expressed as the mean unit ± SEM (*** *p* < 0.001; ** *p* < 0.01; * *p* < 0.05) of three independent experiments in triplicate. Bars represent mean normalized gene expression compared to (**a**) AC-1 DMSO control or (**b**) LTLT-Ca control; (**c**) AC-1, LTLT-Ca and LTLT-Ca cells treated with 10 μM glyceollin I were assayed by immunoblot to examine the expression of ZEB1 and GAPDH (loading control). Representative immunoblot depicts the protein expression of ZEB1 and GAPDH in various cells.

Expression of the nuclear factor ZEB1 has been demonstrated to induce EMT and confer a metastatic phenotype on carcinomas by repressing the E-cadherin gene at the transcriptional level [11,13,14]. Additionally, the ZEB1 transcriptional repressor promotes metastasis through downregulation of microRNAs (miRs) that are strong inducers of epithelial differentiation and inhibitors of stem cell factors [31]. While it is known that miR-200 family members directly target ZEB1, interestingly in a previous report we summarized phytoalexins (including glyceollins) and their emerging role in the regulation of miRNAs and found that the glyceollin mixture did not significantly alter miR-200 family members [23,32]. In order to specifically study the role of glyceollin I on EMT, we attempted to initially identify the mechanism by which glyceollin I alters tumorigenesis and potentially metastasis, by measuring a panel of genes involved in cell motility and EMT (Figure 3a,b). Gene expression studies demonstrate that when compared to letrozole sensitive cells, letrozole-resistant cells expressed 4.51-fold higher levels of ZEB1. However, when LTLT-Ca cells are treated with glyceollin I, ZEB1 expression is reduced by −3.39 fold, demonstrating that glyceollin I can reduce LTLT-Ca ZEB1 expression in LTLT-Ca cells to a level similar to that of letrozole-sensitive cells. Western blot analyses were also performed and ZEB1 protein expression results directly correlated with ZEB1 gene expression (Figure 3c) suggesting that glyceollin I alters critical regulators of EMT in letrozole resistant breast cancer cells. Additionally, gene expression analysis demonstrated that as cells transition from letrozole-sensitive to letrozole resistance there is decreased ESR1 (ERα), TFF1 (pS2) with increased EGFR. However, when LTLT-Ca cells are treated with glyceollin I TFF1 and EGFR levels are decreased, suggesting that glyceollin may regulate LTLT-Ca cells through growth factor and to a lesser extent estrogen signaling.

**Figure 4:**
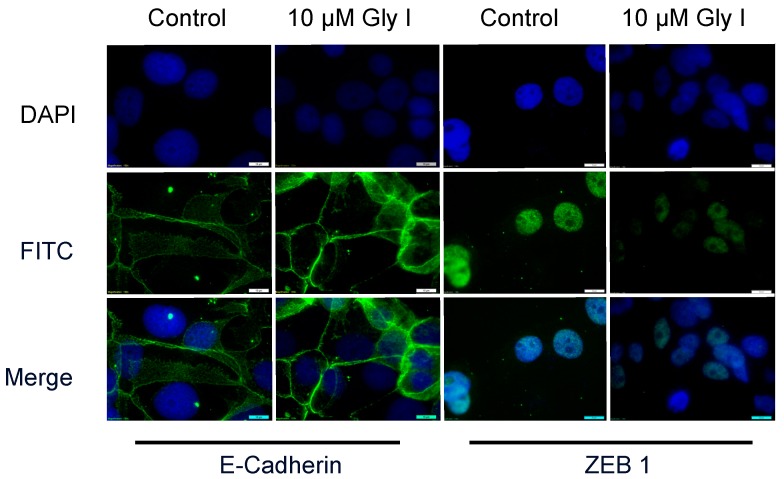
Glyceollin I Induces E-cadherin Protein Expression. Representative immunofluorescence images of LTLT-Ca cells treated with control (DMSO) or 10 μM Glyceollin I for 24 h. Upper panels represent DAPI (blue) nuclear stained control and treated cells; middle panel represents anti-E-cadherin (green) FITC epithelial stained control and treated cells or ZEB1 (green) nuclear stain; bottom panel represents merged images of control and treated LTLT-Ca cells. Original magnification, 100X.

Expression of the nuclear factor ZEB1 induces EMT and confers a metastatic phenotype on carcinomas by repressing the E-cadherin gene at the transcriptional level. E-cadherin is a transmembrane glycoprotein that is responsible for mediating calcium-dependent, homotypic cell-cell adhesion and plays a role in maintaining the normal phenotype and architecture of epithelial cells [13]. Loss of E-cadherin is a critical initial step in the transdifferentiation of epithelial cells to a mesenchymal phenotype, which occurs when tumor epithelial cells invade into surrounding tissues and is associated with metastatic breast cancer [13]. Therefore, since letrozole-resistant cells displayed a higher level of migratory and invasive behavior than the letrozole sensitive cells, and were previously shown to express more mesenchymal markers such as twist and vimentin [9], we chose to further explore their metastatic properties by performing E-cadherin and ZEB1 immnuofluorescence. We compared the expression of E-cadherin and ZEB1 in the presence and absence of glyceollin I. Glyceollin I treatment induced cytoplasmic E-cadherin expression while decreasing nuclear ZEB1 expression (Figure 4). Alteration in the expression of E-cadherin and ZEB1 lend additional evidence in support of the potential of glyceollin to reverse EMT in letrozole resistant breast cancer cells.

### 3.4. Glyceollin Inhibits the EMT-Like Phenotype in Vivo

**Figure 5:**
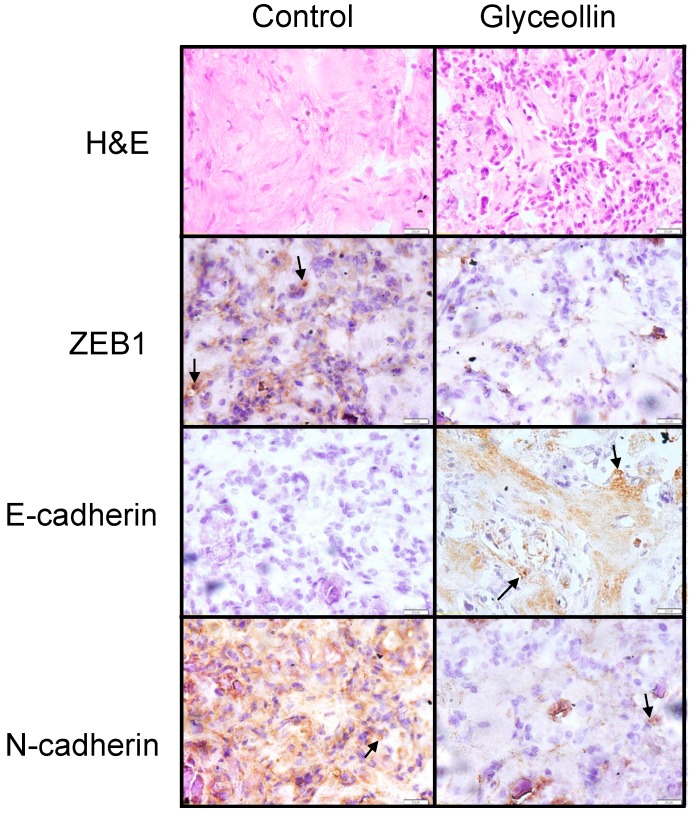
Glyceollin alters EMT Marker Expression *in vivo*. LTLT-Ca cells were injected into the mammary fat pad of 4–6 week old ovariectomized nude female mice. Mice were given glyceollin i.p. at 20 mg/kg/mouse/d for 40 days starting after tumors were measureable. After 41 days, animals were sacrificed and organs removed for further analysis. Representative sections of tumor sections stained with hematoxylin, anti-ZEB1, anti-E-cadherin and anti-N-cadherin. Left panel represents control-treated tumor sections and right panel represents glyceollin-treated tumor sections. Original magnification, 40×. Arrows indicate location of positive staining.

Enhanced cell motility and EMT are positively associated with increased metastatic potential. Our observation of a glyceollin I mediated increase in the epithelial marker E-cadherin as well as a decrease in ZEB1 led us to examine whether the *in vitro* studies translated *in vivo*. Therefore, we examined letrozole-resistant tumor tissue of animals treated with or without glyceollin. Female ovariectomized nude mice injected in the mammary fat pad with LTLT-Ca cells were treated with i.p. injections of glyceollin mixture (20 mg/kg/day) or vehicle control for 40 days. At necropsy, tumors were weighed, harvested and fixed in 10% formalin for 2 days and then processed and embedded in paraffin. Sections were cut and stained for H&E, anti-ZEB1, anti-E-cadherin or anti-N-cadherin (Figure 5) with a DAPI nuclear stain. Our results indicated that glyceollin-treated tumors contained dramatically less ZEB1 expression compared to controls and scored 1 and 3 respectively. Since it has been suggested that alterations in cadherin function may be a critical step in the development of breast cancers we also chose to examine the E- and N-cadherin expression *in vivo*. Likewise, consistent with *in vitro* immunofluorescence staining studies, glyceollin-treated tumors were strongly stained for E-cadherin and scored 3 whilst control tumors stained weakly or not at all for E-cadherin and scored between 0 and 1. N-cadherin staining was also performed and demonstrated an inverse pattern compared to E-cadherin, where control treated tumors were very strongly positive (score 4) and glyceollin-treated tumors had decreased expression (score 1). Interestingly, at necropsy when tumors were weighed, there was no statistically significant difference in tumor weights between the control and glyceollin-treated animals (8.28 ± 0.72 mg and 10.13 ± 1.66 mg, respectively). This result was not surprising since this was a short-term study and certain phytochemicals, such as glyceollins, may require long-term exposure to reduce tumor volume.

### 3.5. Glyceollin I Inhibits Letrozole-Resistant Cell Motility

**Figure 6:**
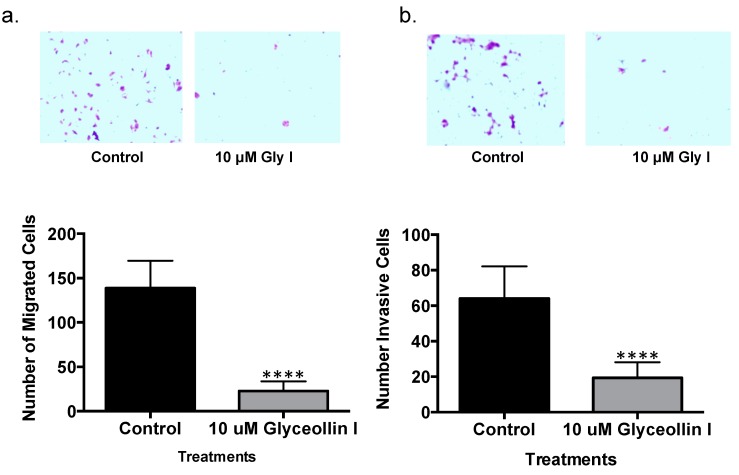
Glyceollin I inhibits the LTLT-Ca cell motility. (**a**) 2.5 × 10^4^ cells were seeded in the upper chamber of a transwell insert (8 μM pore size) alone (migration) or (**b**) coated with Matrigel^®^ (invasion). Cells were treated with 10 μM glyceollin I or vehicle control at time of plating. Lower wells contained IMEM supplemented with 10% CS-FBS. After 24 h, migrated or invaded cells were fixed and stained for visualization. Upper panels are representative images of crystal violet stained migratory cells. Results are expressed as the mean unit ± SEM (**** *p* < 0.0001; *** *p* < 0.001; ** *p* < 0.01; * *p* < 0.05) of four independent experiments in triplicate.

Previous studies led by our group demonstrated that cell motility is enhanced by letrozole resistance which is associated with a more aggressive phenotype [9]. Since glyceollin I altered the letrozole resistant cell morphology to a more epithelial phenotype while inhibiting viability, proliferation and enhancing E-cadeherin expression we were interested in performing proof of concept experiments to determine whether these alterations were associated with a glyceollin I-induced change in cell motility. Our data also demonstrated that glyceollin treatment decreased N-cadherin expression *in vivo* which has been reported to promote motility and invasion in carcinoma cells [33,34]. Taken together this thereby prompted us to perform motility analyses. Migration assays were performed with LTLT-Ca cells treated with DMSO control or glyceollin I for 24 h and seeded in the appropriate serum-free media on the inner chamber of a cell culture insert (for invasion assays a Matrigel-coated cell insert was used). Cells were incubated for 24–48 h and afterwards were fixed and stained. The cells that migrated were imaged and counted. Migration assays revealed that glyceollin I reversed cell migratory behavior by as much as 83% (Figure 6a), while invasion studies demonstrated the potential of glyceollin I to inhibit invasion by more than 68% of the control values (Figure 6b). This therefore demonstrated that glyceollin I can reverse the invasive properties associated with letrozole resistance.

### 3.6. Forced Expression of ZEB1 Suppresses the Effects of Glyceollin I on EMT Markers

**Figure 7:**
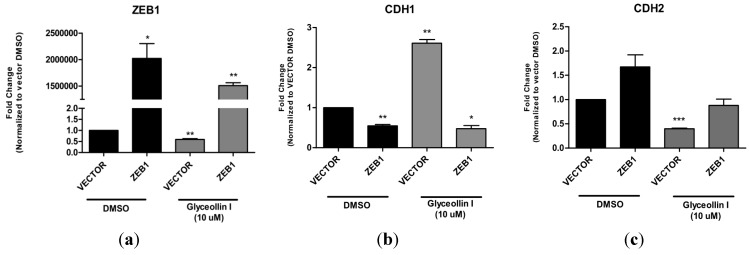
Overexpression of ZEB1 abrogrates the effects of Glyceollin I. Total RNA was isolated from LTLT-Ca vector cells or LTLT-Ca ZEB1 transfected cells, reverse transcribed into cDNA and subjected to quantitative real time RT-PCR analysis. Both LTLT-Ca vector and LTLT-Ca-ZEB1 transfected cells were treated with either DMSO vehicle (as demonstrated by the solid black bars), or 10 μM stimulation with Glyceollin I (as demonstrated by the solid gray bars) and mRNA expression was measured for (**a**) ZEB1; (**b**) CDH1; and (**c**) CDH2. Bars represent mean normalized gene expression compared to vehicle treated LTLT-Ca vector control. Results are expressed as the mean unit ± SEM (*** *p* < 0.001; ** *p* < 0.01; * *p* < 0.05) for three independent experiments.

We found ZEB1 expression levels significantly reduced following glyceollin treatment. Additionally, expression of E-cadherin, a direct target of ZEB1, was significantly elevated by glyceollin I in LTLT-Ca cells. To determine if the effects of glyceollin I on EMT in letrozole resistant cells could be overcome by exogenous overexpression of ZEB1, LTLT-Ca cells were transiently transfected with ZEB1 or empty vector expression plasmids. Following selection, ZEB1 expression was confirmed by qPCR and was found significantly overexpressed. Futhermore, 24 h treatment with glyceollin I significantly reversed the inhibitory effects of glyceollin I on ZEB1 in LTLT-Ca ZEB1 overexpressing cells (Figure 7a). We next measured the effect of ZEB1 overexpression on E-cadherin (CDH1) gene expression. Results demonstrate that in the presence of DMSO, ZEB1 overexpression reduced CDH1 expression (Figure 7b). However, glyceollin I treated empty vector cells resulted in a dramatic induction of CDH1 expression which was suppressed by ZEB1 overexpression. Compared to the DMSO vector control, the glyceollin I treated vector cells resulted in reduced CDH2 expression (Figure 7c). However glyceollin I was not able to significantly suppress CDH2 expression when ZEB1 was overexpressed suggesting that ZEB1 overexpression suppressed the effects of glyceollin I. This therefore implies that by overexpressing ZEB1, glyceollin I loses the ability to convert LTLT-Ca cells to an epithelial phenotype as demonstrated by the lack of induction and repression of CDH1 and CDH2 expression, respectively (Figure 7).

## 4. Discussion

The aromatase inhibitor, letrozole, has been an approved first-line agent for postmenopausal women with locally advanced or metastatic estrogen dependent breast cancer for ten years. The introduction of the aromatase inhibitors made a monumental leap in the field of breast cancer therapy and clinical trials have demonstrated the superiority of letrozole over tamoxifen in first-line endocrine therapy in postmenopausal women with advanced hormone receptor positive breast cancer [35]. Unfortunately, after several years of therapy some patients develop acquired letrozole resistance and are no longer responsive to therapy. As such, there remains a critical need to understand the mechanisms of resistance and develop novel strategies to overcome this problem. While recent studies have demonstrated that histone deacetylase inhibitors such as panobinostat abrogated the growth of aromatase inhibitor-resistant cells *in vitro* and *in vivo*, caused cell cycle G2/M arrest, and induced apoptosis it failed to cause tumor regression [3]. Likewise, etinostat has been demonstrated to slow the growth of letrozole resistant breast cancer cells and has been suggested to restore the responsiveness to letrozole through modulation of Her2, however its effect on metastasis is unclear [8]. Both strategies addressed proliferation, but until recently very little information is available regarding a targeted approach towards reversing and/or suppressing metastasis.

Recently, glyceollins have been described to contain anti-tumorgenic properties in triple-negative breast carcinoma cell systems [23]. Given some similarity between triple negative breast cancers and letrozole resistant breast cancers (*i.e*., both are estrogen-independent and progesterone receptor negative) [9], we sought to explore the inhibitory properties of glyceollins in a letrozole resistant cell line model (LTLT-Ca cells) and demonstrate for the first time the ability of glyceollins to reverse EMT. Previously, we demonstrated that as cells become resistant to letrozole they transition to a more aggressive, estrogen independent and metastatic phenotype. As metastatic breast cancers account for the majority of breast cancer related deaths it is becoming critically more important to develop not only antiproliferative agents but also those that target metastases. Given the antiproliferative effect of glyceollins in hormone-dependent breast tumors and its implication in some hormone refractory breast cancer cells, we tested the hypothesis that glyceollin I exhibits anti-metastatic behavior in letrozole resistant breast cancer cells. First, glyceollin I inhibited the growth and viability of letrozole resistant cells *in vitro* (Figure 1a,b). Since glyceollin treatment did not alter ERα protein expression, but reduced EGFR and ERα gene expression this implied that glyceollin may have both direct and indirect effects mediated through growth factor signaling and estrogen signaling (Figure 2b and Figure 3b). To further assess the properties of glyceollin I in a letrozole refractory cell line, the morphology of the cells were examined pre- and post- glyceollin I treatment. As a result glyceollin I induced a shift in the shape of the cells. Interestingly this epithelial-like morphology closely resembled that of the letrozole-sensitive AC-1 cells, implying that glyceollin I treatment may revert letrozole-resistant cells to a letrozole-sensitive phenotype. While glyceollin I exhibited antiproliferative effects on LTLT-Ca cells *in vitro*, we were not surprised that this response was not recapitulated *in vivo*. It may require extended/chronic exposure before antiproliferative effects are observed with natural products. Therefore, future studies will be required to assess whether long-term glyceollin treatment (greater than 40 days) can alter tumor weight.

While the precise molecular mechanism(s) underlying metastasis remain unclear, the loss of an epithelial phenotype and acquisition to a mesenchymal phenotype has been implicated in cancer cell invasion and dissemination. The ZEB family of transcription factors, which includes ZEB1 and ZEB2, has been demonstrated to mediate this transition by downregulating the expression of genes associated with an epithelial phenotype. The ZEB1 transcription factor is best known as an inducer of EMT in cancer metastasis, acting through transcriptional repression of E-cadherin. When compared to letrozole-sensitive cells, ZEB1 gene and protein expression was induced in LTLT-Ca cells (Figure 3a,b). Interestingly, glyceollin I reduced both the ZEB1 protein and gene expression in LTLT-Ca cells and tumors supporting the hypothesis that glyceollin I is implicated in repressing EMT. To confirm this finding, we tested the expression of E-cadherin, a downstream target of ZEB1 and found that glyceollin I induces E-cadherin protein expression *in vitro* and *in vivo* (Figure 4) further supporting the morphology studies. ZEB1 overexpression studies were conducted to validate the requirement of ZEB1 in glyceollin-mediated reversion of EMT. Our results indicated that ZEB1 overexpression abrogated the inhibitory effects of glyceollin I (as demonstrated in Figure 7) suggesting the effects of glyceollin I are mediated in part by ZEB1. Finally, motility assays were performed and glyceollin I significantly abrogated both migration and invasion demonstrating the dual nature of glyceollins as both an antimetastatic and antiproliferative phytochemical *in vitro* which may prove useful in the management of letrozole-resistant breast cancers. Taken together, we have demonstrated for the first time that glyceollin I induces plasticity among LTLT-Ca cells by shifting EMT to a more epithelial phenotype as demonstrated by enhanced E-cadherin, decreased motility and decreased ZEB1. Elucidation of pathways involved in ZEB1 function is an important step in understanding the processes underlying metastasis and has the potential to yield new therapeutic targets.

## 5. Conclusions

Cancer cells often co-opt many of the normally occurring cellular processes including EMT. As a result cancer cells can exploit the developmental aspects of EMT by capitalizing on epithelial cells losing their cell polarity and cell-cell adhesive interactions to aid in tumor invasion and metastasis. These migratory cells invade surrounding tissue and migrate to distant sites reflecting the cell migration that occurs during normal development. Recent evidence suggests that a small subpopulation of highly tumorigenic cells (*i.e*., cancer stem cells or tumor initiating cells) that are *de novo* radiation- and chemo-resistant are involved in relapse, metastasis, and are implicated in EMT [36]. Furthermore, cells with an EMT phenotype induced by different factors are rich sources for cancer stem cells. As letrozole-resistance is associated with many of the characteristics of EMT, and more recently, cancer stem cells [37], induction of EMT in tumor cells not only promotes invasion and metastasis but also contributes to drug resistance. Future studies will be critical in determining the contribution of cancer stem cells in both acquired and *de novo* letrozole resistance.
